# ADRB1 was identified as a potential biomarker for breast cancer by the co-analysis of tumor mutational burden and immune infiltration

**DOI:** 10.18632/aging.104204

**Published:** 2020-11-21

**Authors:** Jia Wang, Xiaolu Zhang, Jie Li, Xiaoran Ma, Fubin Feng, Lijuan Liu, Jibiao Wu, Changgang Sun

**Affiliations:** 1 College of Traditional Chinese Medicine, Shandong University of Traditional Chinese Medicine, Jinan 250014, Shandong, P. R. China; 2 College of First Clinical Medicine, Shandong University of Traditional Chinese Medicine, Jinan 250014, Shandong, P. R. China; 3 Department of Oncology, Weifang Traditional Chinese Hospital, Weifang 261000, Shandong, P. R. China; 4 Innovative Institute of Chinese Medicine and Pharmacy, Shandong University of Traditional Chinese Medicine, Jinan 250014, Shandong, China

**Keywords:** breast cancer, tumor mutational burden, immune infiltration, ADRB1, prognosis

## Abstract

Breast cancer (BRCA) has traditionally been considered as having poor immunogenicity and is characterized by relatively low tumor mutational burden (TMB). Improving immunogenicity may improve the response to clinical immunotherapy of BRCA. However, the relationship between TMB, immune infiltration, and prognosis in BRCA remains unclear. We aimed to explore their interrelations and potential biomarkers. In this study, based on somatic mutation data of BRCA from The Cancer Genome Atlas (TCGA), patients were categorized into high and low TMB groups utilizing the TMB values. CIBERSOFT algorithm indicated significant infiltration of activated partial immune cells in high TMB group. Besides, ADRB1 had been identified as a prognosis-related immune gene in the mutant genes by the combination of the ImmPort database and the univariate Cox analysis. ADRB1 mutation was associated with lower TMB and manifested a satisfactory clinical prognosis. Various database applications (Gene Set Enrichment Analysis, Tumor IMmune Estimation Resource, Connectivity Map, KnockTF) supported the selection of treatment strategies targeting ADRB1. In conclusion, TMB was not an independent prognostic factor for BRCA and high TMB was more likely to activate a partial immune response. ADRB1 was identified as a potential biomarker and may provide new insights for co-therapy of BRCA.

## INTRODUCTION

Programmed death-1 (PD-1) and programmed death ligand-1 (PD-L1) are immune checkpoint inhibitors (ICIs) [1], which is the most studied type of immunotherapy for breast cancer (BRCA) according to relevant statistics [2]. TMB is a novel marker for evaluating the therapeutic effect of PD-1 antibodies, which has been confirmed in the treatment of colorectal cancer with defects in mismatch repair [3, 4]. It is worth mentioning that TMB may be a promising tumor biomarker [5], defined as the total number of somatic gene coding errors, base substitutions, gene insertions or deletion errors per megabase (Mb). Higher TMB in tumors was reported to facilitate the formation of more new antigens and enhance tumor immunogenicity, which could improve clinical responses to cancer immunotherapy [6]. For example, patients with high TMB had better responses to ICIs and improved survival rates in melanoma, urothelial carcinoma, non-small-cell carcinoma, and bladder cancer [7–10].

BRCA has traditionally been considered as having poor immunogenicity and is characterized by relatively low TMB [11]. However, the immune responses vary substantially between BRCA subtypes. Triple negative breast cancer (TNBC) and HER-2 (+) BRCA are generally more immunogenic than hormone-sensitive BRCA, as reflected in a higher proportion of tumor infiltrating lymphocytes [12]. In addition, luminal B subtypes can be more immunogenic than luminal A tumors among hormone-sensitive BRCA [13]. Allison and Vogelstein have reported a large number of new antigens in breast and bowel cancer tissues, and all cancers have the potential to accumulate new antigens that the immune system can recognize during tumorigenesis [14]. These findings suggest that TMB may play a predictive role in BRCA. Studies have demonstrated that the proliferation rate and the intrinsic subtype of BRCA were associated with TMB [15, 16], whose role in tumor immunogenicity in BRCA is still unclear.

Meanwhile, certain issues remain with TMB. Previous clinical research found that comparing to patients in the low TMB group, not all patients in the high TMB group benefited from ICIs. Specifically, a subset of patients with mutations in the ERBB family (EGFR /ERBB2) and the deletion of specific 3p segments of the chromosome did not respond to ICIs [17]. Cristescu et al. published a study in Science [18], which has some implications for us: simultaneous detection of T cell activity levels and TMB may be a promising strategy. Indeed, positive correlations between mutations or new antigen loads and immune infiltration have been observed in various cancer types [19, 20]. Therefore, we hypothesized that in combination with immune cell groups, TMB as a quantitative indicator of tumor antigenicity may influence the prognosis of BRCA.

In this study, we investigated the association of TMB with gene mutations, immune responses, and prognosis of BRCA in combination with tumor immune infiltration. Using the gene expression profiling data of BRCA from the TCGA database, different gene expressions between high and low TMB groups were compared, and aspects of the clinical characteristics, gene functions and pathways, as well as immune responses were further evaluated. We attempted to elucidate these relationships: different TMB and clinical outcomes, TMB and immune cell populations, immune cells affected by TMB and prognosis. The findings of this study may provide new biomarkers and potential therapy options for BRCA in the future.

## RESULTS

### Somatic mutation landscape in BRCA

**Figure 1:**
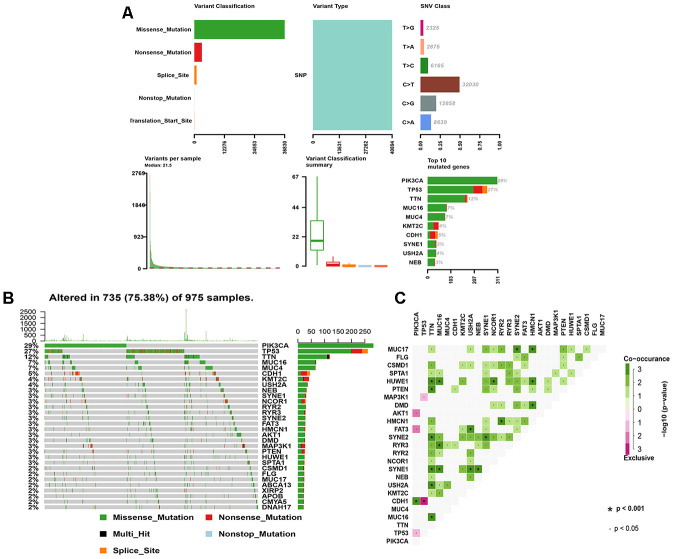
**The landscape of mutation genes in BRCA samples.** (**A**) Classification of mutation types according to different categories, in which missense mutation accounts for the most fraction; SNP appears in all mutations; and C>T is the most common SNV; tumor mutational burden in specific samples; the top 10 mutated genes in BC. (**B**) Mutation information of each gene in each sample is shown in the waterfall plot, in which various colors with annotations at the bottom represent the different mutation types. The bar plot above the legend shows the tumor mutational burden; (**C**) The coincident and exclusive associations across mutated genes. SNP, single nucleotide polymorphism; SNV, single nucleotide variants; BRCA, breast cancer.

Analysis of the 1,044 BRCA mutation samples from TCGA is shown in Figure 1A. Missense mutation was the primary variant classification and all mutations belonged to single nucleotide polymorphisms. C>T was the most common variation in BRCA with the highest number of variations per sample and the median of variation types. In addition, the frequencies of mutations in PIK3CA (29%) and TP53 (27%) were the highest in mutant genes, all of which were missense mutations (Figure 1B). MUC17, HUWE1, SYNE1, TTN, MUC16, HMCN1 had equally higher co-mutation frequencies, while CDH1 and TP53 showed obvious mutuality of mutual exclusion (Figure 1C).

### Correlation analysis of TMB

**Figure 2:**
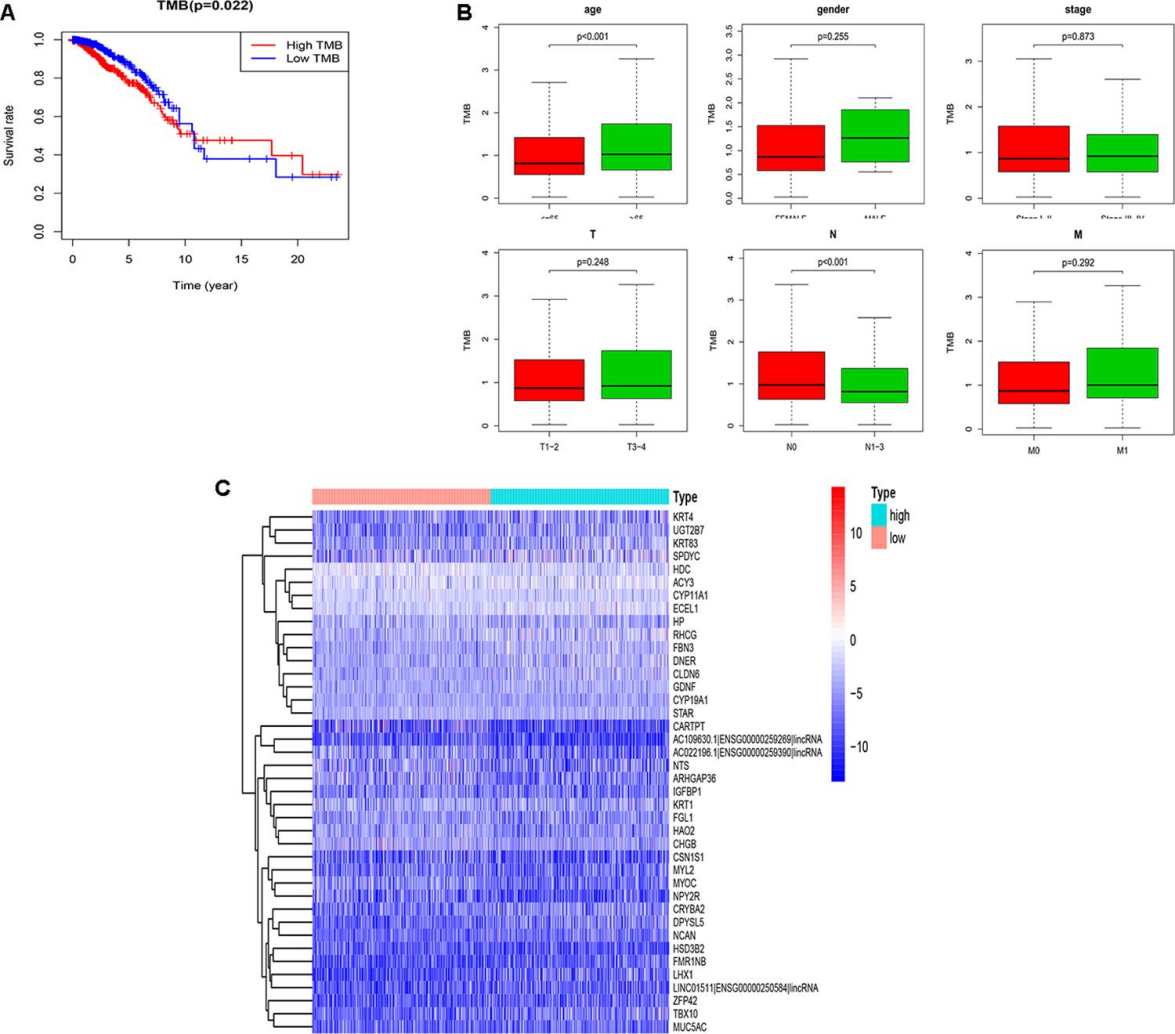
**Performance evaluation of TMB and DEGs in the high and low TMB groups.** (**A**) Prognosis of TMB. The survival curves of the high and low TMB groups intersect (P=0.022); (**B**) The associations of the clinical characteristics with TMB. Higher TMB levels were associated with over 65 years old and the N0 stage (P<0.001); (**C**) The top 40 DEGs are shown in the heatmap plot. TMB, tumor mutation burden; DEGs, differentially expressed genes; N0, no lymph nodes are involved.

The TMB in BRCA ranged from 0.02 to 112.8 per Mb with a median of 0.86 per Mb. With the median TMB value set as the threshold, a total of 986 samples was divided into the high (n=493) and low TMB (n=493) groups. We performed Kaplan-Meier analysis and determined that the 5-year survival rate of was 0.774 for the high TMB group and 0.870 for the low TMB group. Since the high TMB group predicted a better prognosis beyond 10 years, TMB may not be an independent prognostic factor for BRCA (Figure 2A). In addition, among six clinical characteristics, only age and the N stage were significantly correlated with TMB; specifically, patients over 65 years old or with uninvolved regional lymph nodes had higher TMB (Figure 2B). The differential expression of 454 mutant genes between groups is shown in Figure 2C.

### Relationship between TMB and immune infiltration

**Figure 3:**
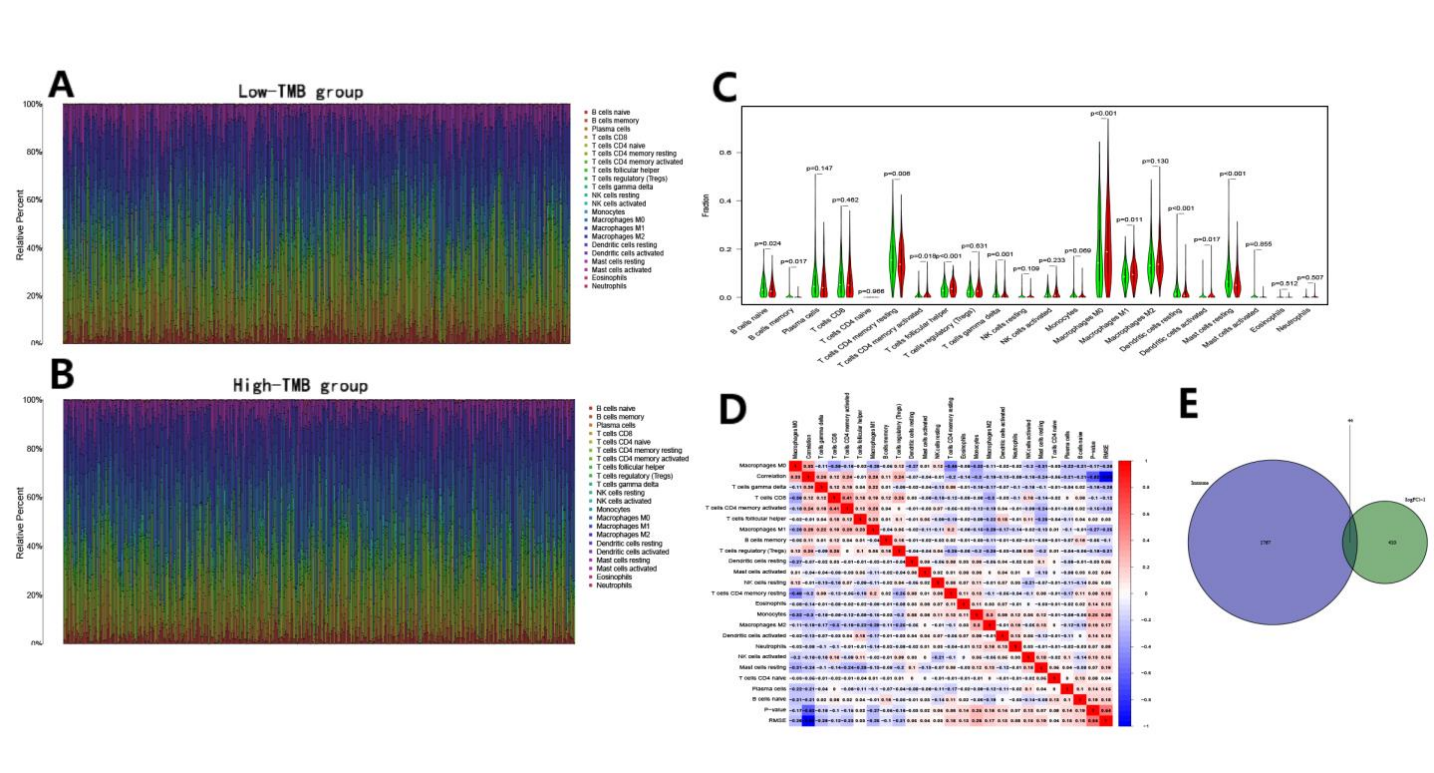
**Immune cell content in the high and low TMB groups and the identification of TMB-related immune genes.** (**A**, **B**) The stacked bar chart indicates the distribution of 22 immune cells in the low and high TMB groups, respectively; (**C**) The violin plot indicates the differentially infiltrated immune cells between in the high and low TMB groups. The green color represents the low TMB group, and the red color represents the high TMB group; (**D**) The correlation matrix of immune cell proportions. The red color represents positive correlations and the blue color represents negative correlations; (**E**) The identification of TMB-related immune genes.

The CIBERSOFT algorithm was used to assess the abundance of immune cells in the high and low TMB groups, and to explore the intrinsic relationship between TMB and the survival rate. Compared to those in the low TMB group (Figure 3A), there were lower levels of B cells and T cells, and higher levels of macrophages in the high TMB group (Figure 3B). Further comparisons indicated that naive/memory B cells, resting CD4^+^ memory T cells, follicular helper T cells, gamma delta T cells, resting dendritic cells, and resting mast cells were abundant in the low TMB group (Figure 3C). For the high TMB group, there were significant infiltration of activated CD4^+^ memory T cells, M0/M1 macrophages, and activated dendritic cells. Furthermore, there were expressional correlations among the subsets of immune cells in transcriptome, a significant negative correlation between M0 macrophages and resting CD4^+^ memory T cells, whereas activated CD8^+^ and CD4^+^ memory T cells were positively correlated (Figure 3D). The Venn diagram showed that 44 immune genes in the differentially expressed genes (DEGs) were screened out (Figure 3E) and ADRB1 was identified as a prognosis-related immune gene by the univariate Cox regression analysis (Table 1).

**Table 1:** Identification of TMB-related immune genes and the univariate Cox regression analysis in BRCA.

**Gene**	**HR**	**HR.95L**	**HR.95H**	**CoxPvalue**
ADRB1*	0.824	0.688	0.987	0.035
SEMA6D*	1.041	1.009	1.074	0.011
FGF14*	1.052	1.006	1.101	0.025
SCG2	1.004	1.002	1.006	9.936
CXCL14	0.999	0.999	1.000	0.320
TMSB15A	1.006	0.992	1.020	0.352
UMODL1	0.966	0.766	1.219	0.775
TNFSF11	0.992	0.951	1.035	0.742
CHGA	0.997	0.990	1.004	0.528
RLN2	1.001	0.986	1.015	0.892
STC2	1.000	0.999	1.001	0.787
TAC1	0.894	0.677	1.179	0.428
ULBP1	1.113	0.977	1.268	0.104
RAET1L	1.074	0.984	1.173	0.107
PDIA2	0.988	0.784	1.274	0.924
SLPI	1.000	0.999	1.000	0.807
LCN2	0.999	0.998	1.001	0.984
S100A9	0.999	0.999	1.000	0.586
S100A8	0.999	0.999	1.000	0.740
MMP12	1.003	0.985	1.020	0.722
PGLYRP4	1.036	0.874	1.229	0.677
FABP6	1.034	0.984	1.087	0.178
MUC5AC	1.003	0.998	1.007	0.145
MARCO	0.992	0.974	1.011	0.430
PCSK1	1.000	0.999	1.001	0.470
VTN	1.003	0.991	1.015	0.557
CCL14	0.898	0.722	1.117	0.336
CMA1	0.995	0.852	1.163	0.958
FGF10	1.001	0.995	1.008	0.641
CX3CR1	0.987	0.941	1.034	0.589
CHGB	1.000	0.999	1.000	0.080
EPO	1.007	0.978	1.037	0.594
GDNF	1.007	0.981	1.032	0.589
GHRH	0.992	0.939	1.047	0.781
NRTN	1.000	0.942	1.062	0.987
NTS	0.988	0.965	1.011	0.307
PTHLH	0.996	0.985	1.008	0.611
SLURP1	1.020	0.993	1.049	0.136
CRLF1	0.994	0.976	1.013	0.557
FGFR4	1.010	0.993	1.027	0.216
IL12RB2	0.955	0.844	1.081	0.472
IL1RL1	1.012	0.909	1.127	0.816
IL22RA2	0.758	0.536	1.071	0.116
PGR	0.996	0.985	1.008	0.583

*, coxPvalue< 0.05.

### Functional enrichment analysis

**Figure 4:**
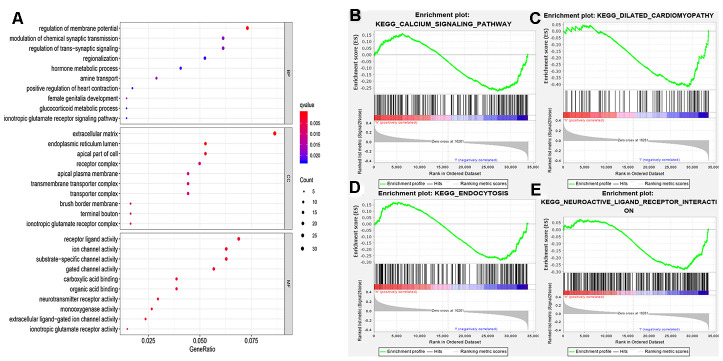
**Functional enrichment analysis.** (**A**) MF, BP, CC in GO categories of DEGs; (**B**–**E**) ADRB1 related pathways using the GSEA software. MF, molecular functions; BP, biological processes; CC, cellular components; GO, gene ontology; DEGs, differentially expressed genes.

We further examined the functional enrichment of DEGs especially ADRB1. Based on gene ontology categories (Figure 4A), ADRB1 was significantly enriched in G-protein coupled receptor binding, neurotransmitter receptor activity, neurotransmitter receptor activity involved in the regulation of postsynaptic membrane potential, and postsynaptic neurotransmitter receptor activity in molecular function, regulation of membrane potential, positive regulation of heart contraction, and heat generation in biological process, synaptic membrane and postsynaptic membrane in cellular component. Gene Set Enrichment Analysis (GSEA) performed with TCGA data indicated that the calcium signaling pathway, dilated cardiomyopathy, endocytosis, and neuroactive ligand-receptor interactions were significantly enriched in samples with ADRB1 (Figure 4B–4E). The findings also showed that the four pathways ADRB1 located in were all significantly active in the low-TMB group.

### CNV of ADRB1, immune cells, and survival in BRCA

**Figure 5:**
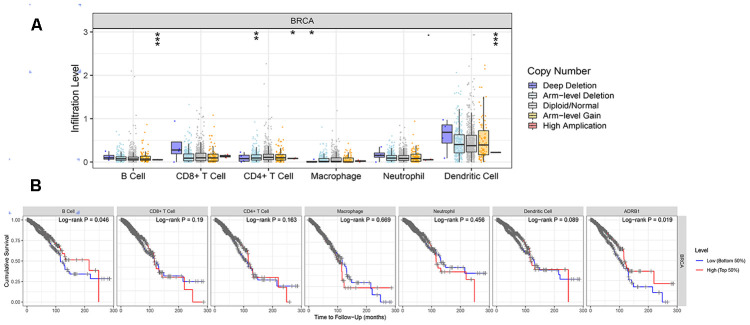
**Correlations between the CNV of ADRB1, immune cell infiltration, and prognosis.** (**A**) High amplification of ADRB1 in B cells and dendritic cells (p<0.001); (**B**) High levels of B cells and ADRB1 suggested better prognosis of BRCA (p<0.05). CNV, copy number variations; BRCA, breast cancer.

Generally, copy number variations (CNVs) refers to the increase or decrease in the copy number of a large segment in the genome whose length exceeds 1 kb. The results were presented in Figure 5A. In B cells and dendritic cells, high amplification of ADRB1 was significantly different compared to other CNVs (p<0.001). In addition, a high level of B cells suggested good prognosis of BRCA, and high expression of ADRB1 may prompt better survival (Figure 5B).

### Various small-molecule drugs of ADRB1 and the transcription factor HIF1A

**Figure 6:**
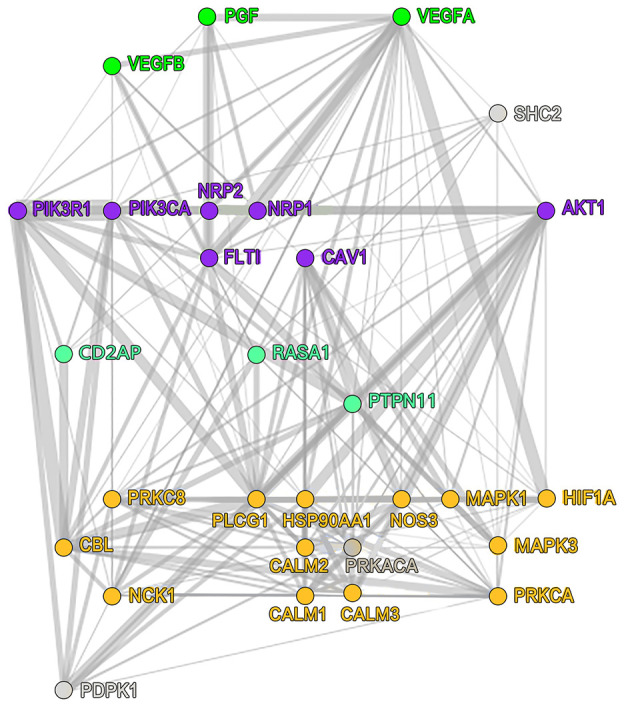
**VEGFR1-specific signaling pathway that HIF1A participates in.** VEGFR1, vascular endothelial growth factor receptor 1; HIF1A, hypoxia-inducible factor 1.

Among the 43 targeted small-molecule drugs predicted for ADRB1, 17 were identified to be acting on BRCA-associated genes (Table 2), including vascular endothelial growth factor A (VEGFR1), dopamine receptor, prolactin, tumor necrosis factor, and polycyclic aromatic hydrocarbons. In the hypoxia inducible factor A (HIF1A) knocking-down dataset in MCF-7 cells (Table 3), the upregulation of ADRB1 maybe not directly regulated by HIF1A, and it might be combined with the AFF1 factor to cause the knock-on effects, AFF1 was detected to bind to the super enhancer and typical enhancer region of the target gene (ADRB1), the regulatory mechanism within is unclear. However, we accidently found that the PIK3CA gene (Figure 6) was involved in the VEGFR1-specific signaling pathway that HIF1A participates in, which has the highest proportion of gene mutations in this study. Its role needs to be further studied.

**Table 2:** Seventeen small-molecule drugs predicted by ADRB1 as well as their effects on BRCA-associated genes.

**Name**	**Target**	**MOA**
amiodarone	KCNH2, ADRB1, CACNA1H, CACNA2D2, CHRM3, CYP2C8, KCNA7, SCN5A	Potassium channel blocker
carvedilol	ADRB1, ADRB2, ADRA1A, ADRA1B, ADRA1D, ADRA2A, ADRA2B, ADRA2C, ADRB3, CYP2C19, CYP2E1, GJA1, HIF1A, KCNH2, NDUFC2, NPPB, RYR2, SELE, VCAM1, VEGFA	Adrenergic receptor antagonist
desipramine	SLC6A2, SLC6A4, ADRA1A, ADRA1B, ADRA1D, ADRA2A, ADRA2B, ADRA2C, ADRB1, ADRB2, CHRM1, CHRM2, CHRM3, CHRM4, CHRM5, DRD2, HRH1, HTR1A, HTR2A, HTR2C, SMPD1	Tricyclic antidepressant
dihydroergocristine	HTR2A, ADRA1A, ADRB1, DRD1, DRD2, DRD3, DRD4, DRD5, HTR1A, HTR3A, HTR4, HTR5A, HTR6, HTR7	Adrenergic receptor antagonist, Prolactin inhibitor
loxapine	DRD2, DRD3, DRD4, DRD1, HRH1, HTR2A, HTR2C, HTR6, ADRA1A, ADRA1B, ADRA2A, ADRA2B, ADRA2C, ADRB1, CHRM1, CHRM2, CHRM3, CHRM4, CHRM5, DRD5, HRH2, HRH4, HTR1A, HTR1B, HTR1D, HTR1E, HTR3A, HTR5A, HTR7, SLC6A2, SLC6A3, SLC6A4	Dopamine receptor antagonist, Dopamine receptor ligand, Serotonin receptor antagonist
mirtazapine	ADRA2A, HTR2A, HTR2C, ADRA2C, HTR3A, ADRA1A, ADRA1B, ADRA1D, ADRA2B, ADRB1, ADRB2, DRD1, DRD2, DRD3, DRD5, HRH1, HRH3, HTR2B, HTR7, OPRK1, SLC6A2, SLC6A3, SLC6A4	Adrenergic receptor antagonist, Serotonin receptor antagonist
sotalol	ADRB1, ADRB2, KCNH2	Adrenergic receptor antagonist
trimipramine	SLC6A2, SLC6A4, SLC6A3, ADRA1A, ADRA1B, ADRA2A, ADRA2B, ADRB1, ADRB2, ADRB3, CHRM1, CHRM2, CHRM3, CHRM4, CHRM5, DRD1, DRD2, DRD5, HRH1, HTR1A, HTR1D, HTR2A, HTR2C, HTR3A	Norepinephrine reuptake inhibitor, Tricyclic antidepressant
amitriptyline	CHRM1, CHRM2, CHRM3, CHRM4, CHRM5, HRH1, HTR6, SLC6A2, SLC6A4, ADRA1A, ADRA1B, ADRA1D, ADRA2A, ADRB1, ADRB2, ADRB3, HRH2, HRH4, HTR1A, HTR1B, HTR1D, HTR2A, HTR2C, HTR7, KCNA1, KCND2, KCND3, KCNQ2, KCNQ3, NTRK1, NTRK2, OPRD1, OPRK1, OPRM1, SIGMAR1	Norepinephrine inhibitor, Norepinephrine reuptake inhibitor, Serotonin receptor antagonist, Serotonin reuptake inhibitor
cabergoline	DRD2, ADRA1A, ADRA2A, ADRA2B, ADRA2C, DRD1, DRD3, DRD4, DRD5, HTR1A, HTR1B, HTR1D, HTR2A, HTR2B, HTR2C, ADRA1B, ADRA1D, ADRB1, ADRB2, HTR7, PRL	Dopamine receptor agonist
nortriptyline	KCNJ10, SLC6A2, SLC6A4, ADRA1A, ADRA1B, ADRA1D, ADRA2A, ADRA2B, ADRA2C, ADRB1, ADRB2, ADRB3, CHRM1, CHRM2, CHRM3, CHRM4, CHRM5, CYP2C19, DRD2, HRH1, HTR1A, HTR2A, HTR2C, HTR6, PGRMC1, PIK3CD, SIGMAR1	Tricyclic antidepressant
propafenone	KCNH2, SCN5A, ADRB1, ADRB2, KCNA5, KCNK2, KCNK3	Antiarrhythmic
pseudoephedrine	ADRA1A, ADRA2A, ADRB1, ADRB2, ATF1, ATF2, ATF3, ATF4, ATF5, ATF6, ATF7, CXCL8, FOS, HRH1, IL2, JDP2, JUN, NFATC1, SLC6A2, SLC6A3, SLC6A4, TNF	Adrenergic receptor agonist
propranolol	ADRB2, ADRB3, ADRB1, CYP2C19, HTR1A, HTR1B	Adrenergic receptor antagonist
olanzapine	DRD2, HTR2A, HTR2C, DRD1, DRD3, DRD4, HRH1, HTR1A, HTR1B, HTR1D, HTR1E, HTR6, HTR7, ADRA1A, ADRA1B, ADRA2A, ADRA2B, ADRA2C, ADRB1, ADRB2, ADRB3, CHRM1, CHRM2, CHRM3, CHRM4, CHRM5, CYP2C8, DRD5, GABRA1, GABRA2, GABRA3, GABRA4, GABRA5, GABRA6, GABRB1, GABRB2, GABRB3, GABRD, GABRE, GABRG1, GABRG2, GABRG3, GABRP, GABRQ, HRH2, HRH4, HTR1F, HTR2B, HTR3A, HTR5A	Dopamine receptor antagonist, Serotonin receptor antagonist
epinephrine	ADRA1A, ADRA1B, ADRA1D, ADRA2A, ADRA2B, ADRA2C, ADRB1, ADRB2, ADRB3, PAH, TNF	carbonic anhydrase activator
norepinephrine	ADRA1A, ADRA1B, ADRA1D, ADRA2A, ADRA2B, ADRA2C, ADRB1, ADRB3, ADRB2, DRD1, DRD5, PAH, SLC18A1, SLC18A2	Adrenergic receptor agonist

**Table 3:** Transcription factors regulating ADRB1 in the breast tissue.

**Target gene**	**TF**	**Knock-method**	**Tissue type**	**Biosample name**	**LogFC**	**Corrected_P**
ADRB1	ELK3	shRNA	Mammary_gland	MDA-MB231	0.68636	5.70000e-04
	PTEN	shRNA		SKBR3	0.89431	6.21560e-01
XBP1	shRNA		MDA-MB231	0.90654	1.24800e-02
	shRNA		T47D	0.96355	1.65000e-03
	HIF1A	siRNA		MCF7	1.09792	6.49070e-01

TF, transcription factor.

## DISCUSSION

TMB was calculated based on the BRCA mutation data from TCGA, and the relationship between the survival curve and TMB showed that TMB may not be an independent prognostic factor for BRCA, which is consistent with previous studies on HER2 (-) metastatic BRCA [21]. We speculated that TMB combined with other prognostic factors may have a better predictive effect. To clarify the internal relationship between TMB and immunologic infiltration, we further showed that the low TMB group had abundant levels of B cells, follicular helper T cells, gamma delta T cells, and various resting immune cells. According to a recent study on triple-negative BRCA [22], the research team used a corresponding single anti CD8^+^ T cells in immune treatment to activate related anti-tumor immune mechanism, while ICIs activated follicular helper T cells that stimulated B cells to produce antibodies. However, the impact on tumor immune responses in inhibiting follicular helper T cells and B cells were more profound than inhibiting CD8^+^ T cells, which demonstrates that B cells and follicular helper T cells play key roles in tumor immune responses. Moreover, higher levels of gamma delta T cells have been shown to be correlated with better outcomes [23]. On the other hand, tumor immunogenicity was enhanced in the high TMB group, leading to significant infiltration of CD4^+^ memory T cells, M0/M1 macrophages, and dendritic cells as well as activated immune responses. The relative increase in TMB was also associated with aging and the N stage, consistent with previous literature that mutations of TP53 in lymph node-negative BRCA were higher than those in lymph node-positive BRCA, and mutations in microtubule-associated proteins may help immune cells recognize tumors and inhibit lymph node metastasis [24].

Furthermore, correlations within the immune cells were determined by analyzing the immune matrix of the entire transcriptome. When investigating the clinical significance of infiltrated immune cells in BRCA, the higher proportion of M0 macrophages indicated a reduced disease-free survival, whereas the increased overall survival was associated with a relatively higher resting CD4+ memory T cells score [25], which corroborates the results of this study. In general, differences in immunogenicity may lead to differences in the activation of immune mechanisms, and the few types of immune cell activation in the high TMB group might indicate that higher TMB suppresses the immune response. It is also worthwhile to note that PIK3CA and TP53 had prominent performance among mutation genes. Previous studies have revealed that mutant allele tumor heterogeneity is positively correlated with TP53 mutation rate, while CDH1 mutation is correlated with a low level of mutant allele tumor heterogeneity [26], confirming the correlation between TP53 and CDH1 observed in this study. The PIK3CA mutation was found in different subtypes such as ER (+), PR (+), HER2 (+), and TNBCs [27, 28], but its role in VEGFR1-specific signaling pathway needs to be further explored. Patients with somatic mutations in TP53 and PIK3CA had reported poor survival [29], and whether the co-mutation of TP53 and PIK3CA can be potential biomarkers for different subtypes of BRCA warrants further investigation.

ADRB1 was eventually identified as a prognosis-related immune gene for BRCA, whose functions were further explored. ADRB1, also called β-1 adrenergic receptor (AR), is a member of the G-protein coupled receptor family and an important target in various therapeutic applications. In cardiomyocytes, proteinkinase A activated by ADRB1 phosphorylates troponin I, the L-type Ca^2+^ channel and phospholamban, while increasing cardiac inotropy, chronotropy, and work [30]. In neuroinflammatory diseases, ADRB1 activation may have neuroprotective effects [31]. Furthermore, experiments have revealed that AR signaling can stimulate the transformation of epithelial cells to mesenchymal cells [32], and ADRB1 was observed to be overexpressed in BRCA tissues [33]. High expression levels of ADRB1 can predict better prognosis in this study, possibly because the overexpression of AR enhances the sensitivity of the tumor to β-blockers, although a previous report claimed that there was no correlation [34]. Future research should further clarify this issue. Pharmacoepidemiologic studies have shown that β-blockers could reduce disease progression and mortality by inhibiting the metastasizing effect of AR signaling [35], but a retrospective analysis indicated that selective β-blockers alone or in combination were less effective than non-selective β-blockers in reducing cell proliferation in BRCA [33]. Interestingly, long-term deprivation of ovarian sex hormones can induce the upregulation of ADRB1 in the heart of rats [36], which suggested that the expression of ADRB1 is up-regulated when the sex hormone shows negative, and in our study, high expression of ADRB1 predicts better prognosis. Therefore, we speculate that HER2 (+) and triple-negative BRCA may be sensitive to β-blockers comparing to sex hormone types (such as the luminal subtype). That is, these two types of breast cancer may be easier to benefit from co-therapy. Prospective clinical trials of β-blockers on various subtypes of BRCA should be the focus of future research.

We further conducted a series of in-depth analyses on ADRB1. High amplification of ADRB1 in B cells and dendritic cells might indicate that ADRB1 mutation can facilitate two types of antigen presenting cells to efficiently mediate and maintain a normal immune response. The better prognosis in patients with high levels of B cells also supports this observation. In addition to CNV, molecular research has demonstrated that the transcription factor HIF1A drives tumor growth and metastasis, and is associated with poor prognosis in BRCA [37]. The inhibition of HIF1A pathway activation combined with β-blockers may be a promising treatment strategy for BRCA patients. In addition, 17 small-molecule drugs targeting ADRB1 and other cancer-related genes obtained in this study also support this proposed treatment.

In conclusion, our study identified prognosis-related immune genes in BRCA mutations based on a co-analysis of TMB and immune infiltration, and explored the intrinsic correlation between TMB and immune infiltration. ADRB1 was identified as a potential biomarker for BRCA, which may provide new insights for co-therapy.

## MATERIALS AND METHODS

### Data collection

Gene expression profiling for BRCA tissue samples (n=109, t=1109) and patients’ clinical data (n=1097) were downloaded from the TCGA portal (https://portal.gdc.cancer.gov/) (Data Release 24.0 -May 07, 2020). In addition, tumor mutation data (n=1044) of BRCA including the names of the mutation genes, the mutation types, and the mutation locations were obtained from the “SomaticSniper variant aggregation and masking” platform.

### Analysis of mutation genes

BRCA samples of TCGA were assessed using the R package “BiocManager” MAF files containing somatic variants and visualized with the maftools package. TMB was obtained by calculating the number of tumor mutations per Mb in each sample. The survival curve was plotted to present the survival rate in relation to TMB. A p-value < 0.05 was considered significant. The limma package was performed to assess the relationship between TMB and clinical characteristics including age, sex, and the stages T, N, and M (p<0.05).

### TMB grouping and differential expression analysis

Normal samples in the tumor mutation data were deleted and the remaining tumor samples were cross-analyzed with the transcriptome samples. The median value of TMB was used as the threshold to divide samples into high and low TMB groups. The DEGs between the two groups were identified using the Wilcoxon rank test. The p value was adjusted by the false discovery rate (FDR) to improve the accuracy of the results, and the thresholds were set as FDR < 0.05 and logFC (fold change) > 1.0.

### Co-analyses of TMB and immune infiltration

The deconvolution algorithm CIBERSORT [38] was used to evaluate the relative abundance of immune cells and the gene expression of tissue samples utilizing the gene expression characterization system of 22 different tumor-infiltrating lymphocyte subsets. The number of permutations was set to 1000, and a p-value <0.05 was regarded as successful. The immune cell matrix was obtained for each sample in the transcriptome data by the CIBERSORT R script v1.03. Similarly, the intrinsic differences in the abundance of immune cells between the high and low TMB groups were further explored and visualized by bar plots. The differences of immune cell infiltration between the high and low TMB groups were visualized by violin plots. Additionally, the list of immunologically relevant genes was downloaded from the ImmPort database (https://www.immport.org/) (Data Release 34, April 2020). Immune genes in DEGs were screened out using the Venn diagram. The univariate cox regression analysis was performed to identify prognosis-related immune genes (p<0.05).

### Functional enrichment analysis

The gene ontology categories including biological processes, molecular functions, and cellular components were assessed for DEGs. Moreover, to determine whether ADRB1-related pathways were statistically and consistently different between the high and low TMB groups, we performed pathway enrichment analysis using the GSEA software (version 4.0.3) with FDR<0.05 considered statistically significant.

### CNV and immune cells

Tumor IMmune Estimation Resource (TIMER v2.0, https://cistrome.shinyapps.io/timer/), a web server for comprehensive analysis of tumor-infiltrating immune cells, was used to estimate the abundances of six immune infiltrates (B cells, CD4^+^ T cells, CD8^+^ T cells, neutrophils, macrophages, and dendritic cells) [39]. Changes in CNV were observed in prognosis-related immune genes, and the correlations between CNV and immune cell abundance, and between immune cells and survival were further assessed.

### Related small-molecule drug prediction and transcription factor signal pathways

Connectivity Map database (CMap, https://clue.io/, data version: 1.1.1.2) [40] was explored to identify small-molecule drug candidates related to BRCA genes. Similarly, KnockTF (http://www.licpathway.net/KnockTF/index.html) [41] was used to comprehensively explore the regulation of gene-related transcription factors as well as signaling pathways with logFC > 1.0.
